# Intratumor Microbiome Analysis Identifies Positive Association Between *Megasphaera* and Survival of Chinese Patients With Pancreatic Ductal Adenocarcinomas

**DOI:** 10.3389/fimmu.2022.785422

**Published:** 2022-01-25

**Authors:** Yu Huang, Ning Zhu, Xing Zheng, Yanhong Liu, Haopeng Lu, Xiaochen Yin, Huaijie Hao, Yan Tan, Dongjie Wang, Han Hu, Yong Liang, Xinxing Li, Zhiqian Hu, Yiming Yin

**Affiliations:** ^1^ Department of General Surgery, No.903 Hospital of People’s Liberation Army Joint Logistic Support Forcel, Hangzhou, China; ^2^ Department of Research and Development, Shenzhen Xbiome Biotech Co. Ltd., Shenzhen, China; ^3^ Shenzhen Institute of Synthetic Biology, Shenzhen Institutes of Advanced Technology, Chinese Academy of Sciences, Shenzhen, China; ^4^ Department of General Surgery, Tongji Hospital, School of Medicine, Tongji University, Shanghai, China; ^5^ Department of General Surgery, Changzheng Hospital, The Second Military Medical University, Shanghai, China

**Keywords:** PDAC (pancreatic ductal adenocarcinoma), intratumor microbiome, 16S rRNA (amplicon sequencing), *Megasphaera*, immune stimulatory

## Abstract

Human tumors harbor a plethora of microbiota. It has been shown that the composition and diversity of intratumor microbiome are significantly associated with the survival of patients with pancreatic ductal adenocarcinoma (PDAC). However, the association in Chinese patients as well as the effect of different microorganisms on inhibiting tumor growth are unclear. In this study, we collected tumor samples resected from long-term and short-term PDAC survivors and performed 16S rRNA amplicon sequencing. We found that the microbiome in samples with different survival time were significantly different, and the differential bacterial composition was associated with the metabolic pathways in the tumor microenvironment. Furthermore, administration of *Megasphaera*, one of the differential bacteria, induced a better tumor growth inhibition effect when combined with the immune checkpoint inhibitor anti-programmed cell death-1 (anti-PD-1) treatment in mice bearing 4T1 tumor. These results indicate that specific intratumor microbiome can enhance the anti-tumor effect in the host, laying a foundation for further clarifying the underlying detailed mechanism.

## Introduction

Pancreatic ductal adenocarcinoma (PDAC) is one of the most lethal cancer types, with a five-year survival rate of around 5% (1). Therefore, treating PDAC represents an unmet medical need. Risk factors for PDAC include smoking, alcoholism, chronic or recurrent pancreatitis, obesity, and diabetes mellitus (2). Recent studies have demonstrated that the microbiome also has a critical role in the progression of PDAC (3, 4). Since intratumor bacteria have been detected in a growing number of tumor types, more attention has been paid to the role of intratumor microbiome in the process of tumor development (5, 6). Intratumor microbiota can promote PDAC progression through inducing innate and adaptive immunosuppression in mice, whereas microbial ablation with antibiotics facilitates T-cell proliferation and immune activation (3). Informative translational studies of the microbiota in PDAC tumor bed combined with preclinical studies based on mouse models have been carried out recently. Follow-up studies have shown that the microbiome of tumor resection samples from short-term PDAC survivors was significantly different from those from long-term survivors, which were partially translocated from the gut and shaped the tumor immune microenvironment (7). In addition to modulating the immune microenvironment, intratumor microbiota can also affect tumor progression through generating metabolic products (8). Moreover, intratumor microbiome of pancreas can originate from the gut microbes through the circulatory system or the biliary/pancreatic duct and gastrointestinal microbiota are susceptible to environmental, diet, and lifestyle factors (9, 10). In addition, the host genetics can also shape gut microbiome (11). Therefore, it is urgently necessary to understand pancreatic tumor microbiome and the impact of their metabolic products on PDAC prognosis in Chinese cohort. On the other hand, tumor immunotherapy studies have been conducted in recent years targeting programmed cell death-1 (PD-1) and programmed death-ligand 1 (PD-L1). Tumor immunotherapy works by activating the immune system. Immunotherapy drugs such as anti-PD-1 increase the ability of immune cells in identifying and attacking cancer cells. However, the immunotherapy response rate in patients with different tumors or at different disease states is inconsistent, and many patients even had no response. PDAC is characterized by low Tumor Mutational Burden (TMB) due to limited expression of neoantigens, leading to the poor response to immunotherapy. Only a small subset (1%-3%) of PDAC patients with deficient Mis Match Repair (dMMR) or microsatellite instability-high (MSI-H) tumors showed high response rate to single agent pembrolizumab (12). Meanwhile, multiple studies have reported that microbiome or their metabolites can enhance the anti-tumor effect of PD1 to a certain extent (13, 14), and tumor growth can be inhibited by injecting modified bacteria that continuously express arginine into the tumor microenvironment (15). Therefore, dominant bacteria identified in the long-term tumor survivors might have influences on the tumor microenvironment and contribute to a better prognosis. Based on these, we collected the PDAC tumor samples from Shanghai Changzheng Hospital, and investigated the microbiome composition in different survival patients. The results showed that the relative abundance of specific bacteria such as *Megasphaera* has a positive correlation with survival time. We further investigated the effect of *Megasphaera* in the PD1-low-response tumor-bearing mouse model and found that *Megasphaera* can significantly improve the anti-tumor efficacy of anti-PD1 treatment. The assessment of PDAC microbiome at the initiation time of PDAC therapy may offer an insight into the prognosis of PDAC patients and the improvement of patient survival through modulating pancreatic and/or gut microbiome.

## Materials and Methods

### Genomic DNA Extraction and Bacteria 16S rRNA Sequencing

A total of 8-10 sections (8 μm) of formalin-fixed, paraffin-embedded (FFPE) tumor tissues from PDAC patients were aseptically collected and placed in 1.5 mL Eppendorf tubes. Paraffin without tissue and nuclease-free water were used as negative controls. Genomic DNA was extracted with QIAamp DNA FFPE Tissue Kit (NO.56404) following the manual. The final product was then eluted with TE buffer and amplified with the primer FWD/REV used in a previous study (7). The obtained 16S V4 region sequence was used for library construction with a second pair of primers containing adapters for sequencing and unique barcoding. All the samples were then pooled and sequenced on a MiSeq Platform following the 2 × 250 bp paired-end protocol. A 30% standard library PhiX was used as a balanced library.

### Raw Data Analysis and Taxonomic Profiling Pipeline

The output reads were demultiplexed based on the unique barcodes, and the primers were removed from the raw reads with Cutadapt v1.18 (16). Sequences with low-quality scores (Q<30) were removed, and forward and reverse reads were trimmed and merged. Chimeras were identified and removed. The quality-filtered and denoised reads were then used to generate amplicon sequencing variants (ASV), the essentially operational taxonomic units (OTU) with 100% identity, with DADA2 (17). Each unique ASV was assigned to a high-resolution taxonomy using the Ribosomal Database Project classifiers (implemented in DADA2) and SILVA Database v132 (18). All the sequencing data were deposited at NCBI under the accession number PRJNA764032.

### Phylogenetic Diversity and Composition Analysis

The resulting contaminant-free 16S rRNA reads were used for calculating relative abundance and the following comparison analyses. The genus-level relative abundance was calculated by summing up the relative abundances of ASVs belonging to each genus on the phylogenic tree. Alpha and beta diversities were computed utilizing the R package Phyloseq (19). For alpha diversity, the Chao1 index and Shannon index were used to represent the richness and evenness of each sample, respectively. Principal coordinates analysis was performed using pair-wise Bray-Curtis dissimilarity to measure the distance among different samples. The statistical differences of alpha diversity across different groups were calculated with the Wilcoxon test.

### Differential Abundance Analysis and Functional Prediction

Differential abundant taxa were identified with DESeq2 (20), Wilcoxon’s rank sum test, and Omnibus (21). The false discovery rate (FDR) was used to correct for multiple hypothesis testing (22). Only those identified by all the three pipelines were considered as differential genera. Linear discriminant analysis Effect Size was used for linear discriminant analysis. Differentially abundant genera were used for heatmap visualization. The contaminant removal 16S rRNA ASV sequences were then used for functional gene content by PICRUSts (phylogenetic investigation of communities by reconstruction of unobserved states), a technique which uses evolutionary modeling to predict metagenomes from 16S data and reference genome databases. Predicted metagenomes were then used as inputs for metabolic reconstruction, using level-3 KEGG pathways to predict the function of the intra-tumor bacterial community (23). Differential KEGG categories that were identified by DEseq2 and Omnibus were plotted and colored according to enrichment or depletion between LTS and STS groups (KEGG level 3, FDR adj.p.vaule<=0.01, and |log_2_ fold change|>=1).

### qPCR Validation

Bacteria DNA was extracted from frozen PDAC tissue samples with QIAamp DNA Microbiome Kit (cat # 51704). qPCR was performed with primers specific to *Sphingomonas* (Sph–spt 694f:GAGATCGTCCGCTTCCGC, Sph–spt 983r:CCGACCGATTTGGAGAAG) and *Megasphaera* (XA511-qF1: ACTACCTGCCCTCTGCCGATAA, XA511-qR1: CGAGCATAGCGGTCCCATTGAA). The 16S universal primer (Uni-qPCR-F: AGAGTTTGATCCTGGCTCAG, Uni-qPCR-R: TGCTGCCTCCCGTAGGAG) was also used to quantify the total bacteria. Relative abundance of *Sphingomonas* as well as *Megasphaera* was calculated with the 2^-ΔΔ^ *^CT^* method (24).

### Short-Chain Fatty Acids (SCFA) Measurement

The composition and expression levels of SCFAs in the supernatant fraction of different bacteria were determined by GC/MS (8890-7000D, Agilent Technologies) with an HP-5 ms capillary column (30 m × 0.25 mm × 0.25 μm film thickness) (Agilent Technologies). Samples were acidified with HCl and extracted with anhydrous diethyl ether (1:1, v/v) before processing for the GC/MS analysis. The calibration curve was constructed by plotting relative peak area of each SCFA (peak area of SCFA was normalized to peak area of internal standard) to the corresponding concentration of SCFA internal standard.

### Bacterial Culture and Preparation of Bacterial Fractions

*Megasphaera* sp.*XA511* strain isolated from the stool sample of a healthy volunteer was routinely cultured anaerobically in the modified ATCC 2107 medium. Late log phase bacterial cultures were centrifuged at 5000 rpm for 10 min at room temperature, and the cell pellets were washed once in phosphate-buffered saline (PBS) and resuspended in antibiotic-free cell culture media to the appropriate concentration (live fraction, *Megasphaera* sp.*XA511* LV). The cultural supernatants were harvested and filtered through a 0.22 μm filter to obtain the supernatant fraction (*Megasphaera* sp.*XA511* SN). The bacteria were inactivated at 110°C for 15 min and resuspended as heat-killed fraction (*Megasphaera* sp.*XA511* HK). Viable cell counts were determined by spread plating.

### Co-Culture Assay and Quantification of Cytokines

THP-1 cells were purchased from the National Collection of Authenticated Cell Cultures (China). Human peripheral blood mononuclear cells (PBMCs) were purchased from Shanghai Milestone^®^ Biotechnologies Company. The co-culture assay was performed according to the method of a previous report (25). For THP-1 co-culture assay, THP-1 cells were routinely grown in RPMI 1640 medium supplemented with 10% (v/v) fetal bovine serum (FBS) and 0.05 mM beta-mercaptoethanol (Sigma-Aldrich) and 1% penicillin and streptomycin. Then cells were seeded in 96-well plates (300000 cells/well) and differentiated into macrophages by adding 5 ng/mL phorbol 12-myristate 13-acetate (PMA) (Sigma-Aldrich) for 24 h incubation, followed by washing with PBS and replacing with Complete Medium (CM). After 72 h culture at 37°C in 5% CO_2_, the cells were treated with bacteria fractions (heat-killed fraction at a multiplicity of infection (MOI) of 1:1 or supernatant fraction at an MOI of 10:1) or controls (1 mg/mL lipopolysaccharide (LPS) or PBS) for 24 h. The supernatant was collected for cytokine determination. For live fraction, the cells were incubated with them at an MOI of 1:1 for 2 h under anaerobic conditions and removed the bacteria fraction by centrifugation. Then the cells were washed with PBS and cultured in fresh medium with 100 U/mL penicillin and 100 μg/mL streptomycin for 22 h at 37°C in 5% CO_2_. Cell-free supernatants were then harvested and stored at −80°C for cytokines detection. For PBMCs co-culture assay, cells were recovered in RPMI 1640 medium supplemented with 10% (v/v) fetal bovine serum (FBS). Then the cells were co-cultured with the heat-killed and live bacteria for 2 hours at an MOI of 10:1 or controls under anaerobic condition. The bacteria fraction was removed by centrifuge, cells were continuously cultured in CM with penicillin, streptomycin, and gentamicin for 22 hours. The supernatant was collected by 400g centrifugation, and high-speed centrifugation of 13000g were used to remove the debris, the final supernatant was used for cytokines determination. The expression levels of different cytokines including IL-6, IL-10, IFN10, IFN-γ and TNF-α were determined using the LEGENDplex™ HU Th Cytokine Panel (12-plex) w/VbP V02 kit following the manufacturer’s recommendations (Biolegend).

### Animal Experiment

The animal experiment was performed in Shenzhen TOP Biotechnology Company following approval by the Institutional Animal Care and Use Committee of the company. Six-week-old specific pathogen-free BALB/c female mice were used in this study. A total of 40 mices were equally divided into four groups randomly,which were Control, *Megasphaera* sp.*XA511*, anti-PD1 and *Megasphaera* sp.*XA511* combined with anti-PD1 groups.The 4T1 cells purchased from China Center for Type Culture Collection were cultured at 37°C and 5% CO_2_ in complete DMEM supplemented with 10% FBS, 100 U/mL penicillin, and 100 µg/mL streptomycin. The mice were subcutaneously injected with 100 µL of the cell suspension (1×10^6^ cells/mL) into the right flank. After tumor inoculation, the tumor-bearing mice were daily administrated with 2×10^9^ CFU bacteria by gavage throughout the experimental period. Once the tumors were established at roughly 25-100 mm^3^, tumor volume was measured every other day. Digital calipers were used to measure the length and width of each tumor on day7, day9, day11, day14, day16, day18 and day21 after tumor inoculation. Tumor volume was calculated as length × width^2^ × 0.5, where the width was the smaller one of the two measurements. For anti-PD1 or anti-PD1 combined with *Megasphaera* sp.*XA511* groups, the anti-PD-1 antibody (Clone RMP1-14, BioXcell) or isotype control was administered to the mice on days 9, 12, 15, and 18 by intraperitoneal injection (100 µg/injection).

### Multiplex Panel Immunofluorescence (mIHC)

Multiplex panel immunofluorescence (mIHC) staining of tumor tissues was performed by Crown Bioscience Inc. 5 samples from each of the four groups were first used for paraffin embedding, and the FFPE samples were then used for mIHC experiment. Briefly, 4 μm thickness/sections of FFPE tumor cross section tissues were mounted on slides. Then, the slides were deparaffinized in xylene and rehydrated in graded ethanol. Anti-mouse antibodies against the following: CD3 (Invitrogen, Cat# MA5-14524), CD4 (Cell Signaling,Cat# 25229), CD8 (Cell Signaling, Cat#98941), CD11c (Cell Signaling, Cat#97585), IFN-γ (Abcam, Cat#ab216642) were used with the Bond RX autostainer for Multiplex staining. All stained sections were scanned with Vectra^®^ multiplexed imaging systems at 20x magnification. High resolution imagery of whole sections was generated and subjected to quantification analysis with HALOTM software. The intensity of nuclear or cytoplasmic positive staining was counted. The positive cells/area were evaluated as follow: Positive cells/area = Positive cells/Tumor Area.

### Statistical Analyses

The patients’ demographic and clinical information in this study was compared using chi-square test. Fisher’s exact test and Wilcoxon’s rank sum test were used to evaluate the association between two categorical variables and to compare the distributions of continuous variables between two different groups. Wilcoxon’s rank sum test was also used to test the Alpha diversity and Bray-Curtis between LTS and STS patients. Kaplan-Meier curve was used for the survival analysis. The Log-rank test was used to test the difference in survival distributions between groups. P<0.05 was considered statistically significant. For qPCR validation, t-test was used for statistical analyses. For the *in vitro* co- culture assay, the one-way analysis of variance (ANOVA) was used to determine whether there are any statistically significant differences between the different bacteria and control group. For the *in vivo* experiment and mIHC, tumor size data were analyzed and expressed as the mean ± standard deviation using ggplot2 package of R studio (v3.6.3). The one-way ANOVA was used to determine whether there are any statistically significant differences between the means of anti-PD1 combined with *Megasphaera* sp. *XA511* and other groups.

## Results

### Characteristics of the Study Cohort

To investigate the impact of microbiome on the progression of PDAC in the Chinese population, FFPE samples from PDAC patients surgically resected between 2014 and 2017 were collected from Shanghai Changzheng Hospital and used for microbiome analysis (**Table 1**). According to the postoperative follow-up data and patient prescription information, the patients who were treated with antibiotics or probiotics 1 month before surgery or neoadjuvant therapy were excluded. All the patients received adjuvant treatment based on gemcitabine in combination with capecitabine after surgery. The patients with an overall survival time of longer than 600 days from surgery were defined as long-term survivors (LTSs), and those with 120 to 300 days from surgery were defined as short-term survivors (STSs). The patients with a survival time of shorter than 120 days were removed from the analysis to exclude perioperative mortalities. The patients in LTS and STS groups were matched with respect to age, gender, and stage. There is a total of 13 STS and 17 LTS patients, including 17 females and 13 males, whose median age was 65, ranging from 46 to 88 years old. Among them, 16 patients were classified as early-stage (all at stage II, and none at stage I), 12 were classified as advance stage (8 at stage II-III, 4 at stage III, and none at stage IV), while the stage information was unknown for 2 patients. There was no statistical difference in the age, gender, or cancer stage between LTS and STS patients by Fisher test (**Table 1**).

**Table 1:** The baseline characteristics of the clinical samples.

Patients Characteristics	STS (n=13)	LTS (n=17)	p value
Survival time (days)	208	708	<0.0001
Surgery date	2014-2017	2014-2016	
**Gender**			
Female	7	10	1
Male	6	7	
**Age (years)**			
Median	63	67	0.966
Range	51-84	46-88	
**Stage**			
II	5	11	0.575
II-III	5	3	
III	2	2	
NA	1	1	

### Significantly Different Intratumor Microbiome Between LTSs and STSs

**Figure 1:**
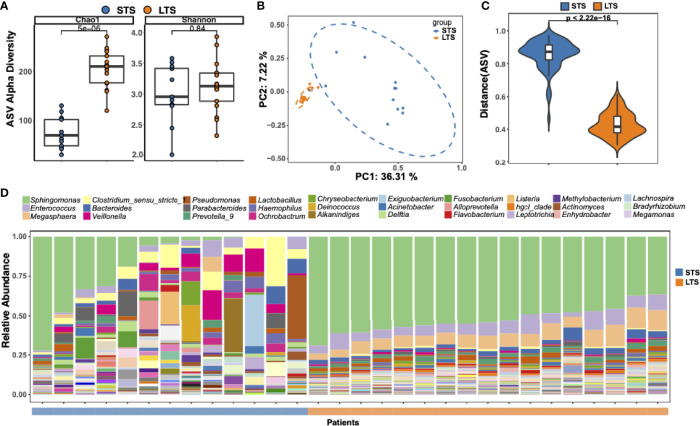
Tumor microbial community differs between long-term and short-term PDAC survivors. **(A)** Alpha diversity boxplot (Chao 1 and Shannon indices) in long-term and short-term survival patients. **(B)** Principal coordinate analysis of different samples using Bray-Curtis metric distances of beta diversity. Orange dot indicates LTS sample, and blue dot indicates STS sample. **(C)** Violin plots of the Bray-Curtis distance between samples from the same group across different patients. **(D)** Stack bar charts of genus-level bacterial composition for the LTS and STS patient samples. Relative abundances were calculated by summing up the reads of species that past all filters and belong to the same genus. The top 30 genera name are shown in the legend.

DNA was extracted from the samples and 16S rRNA sequencing was performed. An average sequencing depth of 62524 with a minimal sequencing depth of 31611 was obtained. ASVs were generated with DADA2. To eliminate any potential effect of contamination, we performed the same extraction and sequencing procedures on 2 paraffin-only samples from the margins of the paraffin blocks and 2 nuclease-free water samples, and the obtained ASVs were removed from further analysis. Although no significant difference in Shannon index was observed between LTSs and STSs, the Chao1 index in LTSs was higher than that in STSs (**Figure 1A**), indicating LTSs had a more diverse intratumor microbiome than STSs, which is in consistent with a previous finding that high microbial diversity is related to better clinical outcomes (7, 19). Principal Coordinate Analysis revealed that the samples from LTS patients were tightly clustered (**Figures 1B, C**), indicating that LTS samples shared a similar microbiome structure. The Bray-Curtis distance between LTS and STS groups was significantly different, indicating that LTS and STS had distinct intratumor microbiome compositions. Therefore, the diversity and composition of the intratumor microbiome are significantly different between LTS and STS patients.

To better understand the difference in microbiota between LTSs and STSs, ASV enrichment was compared at different taxonomy levels (**Supplementary Figure 1**). The major microbial phylum compositions were *Proteobacteria*, *Firmicutes*, and *Bacteroidetes* (**Supplementary Figure 1**), which is in agreement with a previous report (5). Three dominant genera were detected among all the LTS samples, including *Sphingomonas*, *Enterococcus*, and *Megasphaera*, while only few STS samples contained *Sphingomonas*. *Sphingomonas* has also been detected in PDAC patients in previous reports (7, 26, 27). Notably, *Megasphaera* was specifically enriched in the LTS samples. Meanwhile, the abundance of *Clostridium*, which is considered as an opportunistic pathogen (28) in most cases, was higher in STS samples than in LTS samples (**Figure 1D**).

**Figure 2:**
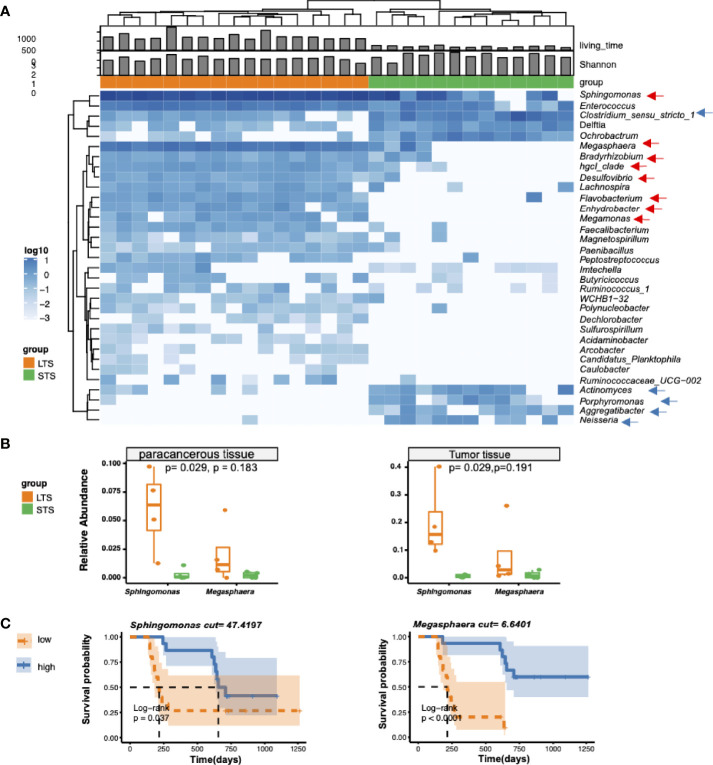
Differential genera and survival analysis. **(A)** Heatmap of differentially bacterial abundant features at the genus level. Blue color represents the highest abundance, lighter blue color represents intermediate abundance, and white color represents lower abundance. Three taxa enriched in LTS samples, and 4 taxa enriched in STS samples (p<=0.001 and |R^2^|>=0.7) are presented. **(B)** Validation of the presence of *Sphingomonas* and *Megasphaera* in tumor tissue from LTS and STS by qPCR. 2^-ΔΔCT^ method was used to calculate the relative abundance. *t*-test was used for statistical significance analysis. **(C)** Kaplan-Meier estimates for survival probability with high abundance versus low abundance of different microbiome in STS and LTS patients.

To clarify the characteristic signal of the LTS and STS samples, differential enrichment analysis was performed with Deseq2, Wilcoxon’s test, and Omnibus. A total of 33 differentially enriched genera were identified by all these 3 methods between LTS and STS samples, including 25 in LTSs and 8 in STSs (**Figure 2A**). LTS samples exhibited higher abundances of *Sphingomonas*, *Megasphaera*, *Bradyrhizobium*, *hgcI_clade*, *Desulfovibrio*, *Flavobacterium*, *Enhydrobacter*, and *Megamonas*, while STS samples exhibited higher abundances of *Clostridium_sensu stricto 1*, *Actinomyces*, *Porphyromonas*, *Aggregatibacter*, and *Neisseria.* Interestingly, a previous study also reported that *Aggregatibacter actinomycetemcomitans* was associated with a higher risk of PDAC (29). Meanwhile, previous research has also shown that the combination of Saliva-derived *Neisseria elongata* and *Streptococcus mitis* can distinguish PDAC patients from healthy individuals, with 96.4% sensitivity and 82.1% specificity (30). In addition, although *Sphingomonas* and *Megasphaera* were not the differential genera by Erick’s study, they were present in the intratumor microenvironment of both LTS and STS patients (7). To further confirm the presence of the dominant bacteria, another 8 frozen tumor tissue as well as paracancerous tissue samples resected from PDAC LTS and STS patients were analyzed by qPCR with primers targetting *Sphingmonas* and *Megasphaera* (31). Indeed, the qPCR assay confirmed that the tumor tissues from LTS patients showed higher abundance of *Sphingomonas* and *Megasphaera* than those from STS (**Figure 2B**). The results also revealed that the relative abundance of bacteria in the tumor tissue is higher than that in paracancerous tissue. (**Supplementary Figure 2A**).

To extend our understanding on the connection between microbiome diversity and patient survival, we investigated the association between the relative microbial abundance and the overall survival by stratifying the patients into two groups based on the median values of the relative abundance of specific genera. As expected, we found that the patients with high relative abundances of *Sphingomonas* and *Megasphaera* were associated with significantly prolonged overall survival (**Figure 2C**), while those with a high abundance of *Clostridium* were associated with shortened survival time (**Supplementary Figure 2B**). However other clinical parameters such as age, CA199, and hemoglobin were not significantly correlated with the differential genera (**Supplementary Figure 3**).

### Metabolic Pathways Associated With Microbial Communities From LTS and STS Patients

**Figure 3:**
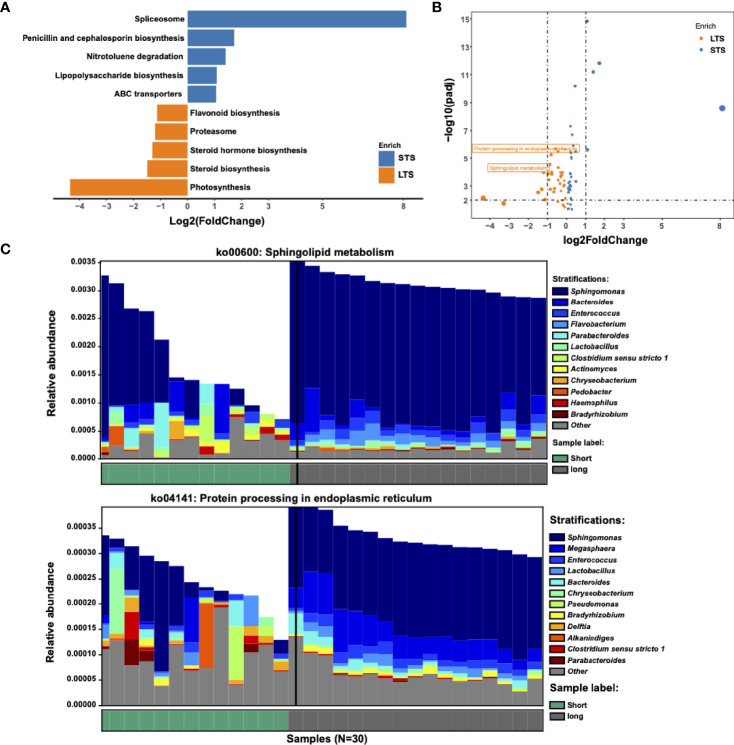
Metabolic pathways driven by microbial communities from LTS and STS patients. **(A)** KEGG pathway analysis was performed. Significantly differential KEGG pathways (p<=0.01 and |log_2_ fold change|>=1) are presented. For each KEGG pathway, the bar shows the fold-enrichment of the pathway. **(B)** Volcano plot of KEGG profiles between LTS and STS patients. **(C)** Contribution of bacterial species to 2 different pathways in 13 STS and 17 LTS patients.

Several studies have shown that microbiome can change tumor microenvironment through producing metabolites (13, 32). Based on this, we speculated if the tumor microbiome could enhance tumor immunity and/or affect the prognosis of the patients through specific metabolic pathways. Differential KEGG pathway analysis identified 74 differential pathways related to 18 core functional modules, including diverse metabolism, cellular processes, and genetic information processing (**Supplementary Table 1**). For example, the sphingolipid metabolism pathway was enriched in LTS samples, which might be attributed to *Sphingomonas* and *Enterococcus*; protein processing in the endoplasmic reticulum, which belongs to the genetic information processing pathway, might be attributed to *Sphingomonas* and *Megasphaera* (**Figure 3**). Microbiome set enrichment analysis (33) was also performed to explore the relationship between differential genera and human diseases. Our results showed that most of the differential genera were involved in tumor immunity pathways, such as IL-17 and TNF signaling (34, 35) (**Supplementary Figure 4**). All these findings suggest that intratumor microbiome may affect the survival probability of patients through changing tumor microenvironment.

### *Megasphaera* sp.*XA511* Stimulates the Secretion of Different Cytokines *In Vitro*

**Figure 4:**
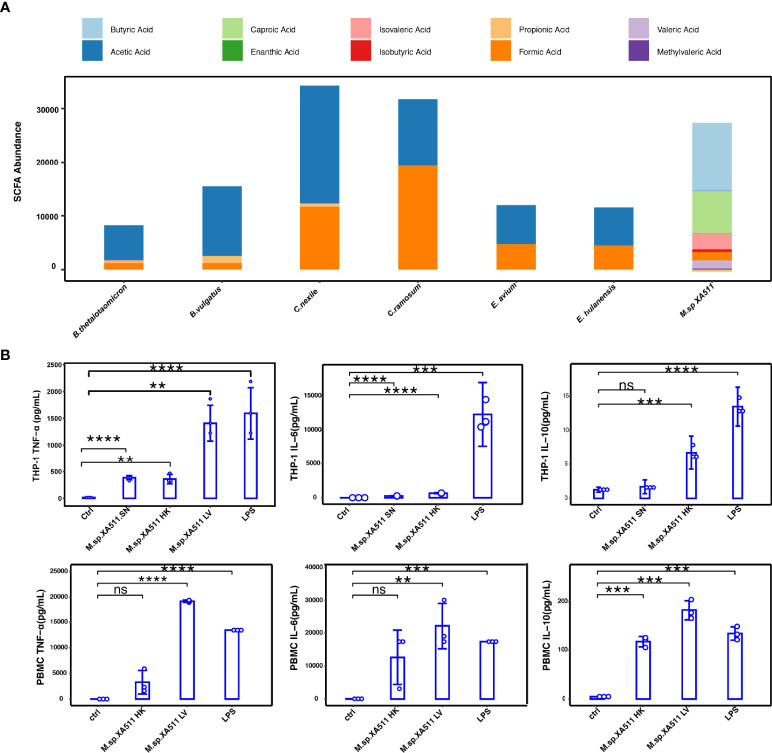
SCFA production of *Megasphaera* sp.*XA511* and effects of *Megasphaera* sp. *XA511* on producing proinflammatory cytokines. **(A)** The production of different SCFAs by *Megasphaera* sp.*XA511* (*M.sp.XA511*) and other bacteria (*B.thetaiotaomicron*, *B.vulgatus*,*C.ramosum*, *C.nexile*, *E.hulanensis* and *E.avium*) were measured by GC-MS. The bacteria were grown until the Late log phase before the measurement of fatty acids in supernatants. **(B)** The secretion of cytokines from THP-1 and PBMCs treated with supernatant derived from *Megasphaera* sp.*XA511* (*M.sp.XA511*-SN) or the heat killing (*M.sp.XA511*-HK) or live (*M.sp.XA511-*LV) fraction was determined. The one-way ANOVA analysis was used to determine whether there are any statistically significant differences between the different bacteria and control group. NS indicates not significant (p > 0.05), whereas the asterisk indicates significant difference (**P < 0.01; ***P < 0.001; ****P < 0.0001).

Consistent with a previous report (7), we have identified that bacterial species were associated with survival in PDAC patients. However, the direct effect of these bacteria in tumor treatment has not been assessed. In this study, we examined the anti-tumor effect of *Megasphaera*, which was enriched in LTSs in our cohort (**Figure 2**). Previous study have indicated that *Megasphaera* species could produce SCFA and enhance the function of cytotoxic T lymphocytes through producing SCFAs (36–38). Therefore, we isolated a *Megasphaera* strain (hereafter termed as *Megasphaera* sp.*XA511*) from the stool of a healthy human donor, and the identity of the strain was confirmed by whole-genome sequencing. Through GC/MS, we confirmed that *Megasphaera* sp.*XA511* could produce a large amount of butyric acid, valeric acid, and isovaleric acid (**Figure 4A**). We also checked the SCFA production levels of two species of *Clostridium* (*C.ramosum* and *C.nexile*) and two species of *Enterococcus* (*E.hulanensis* and *E.avium*), which were enriched in STS, as well as two species of *Bacteroides* (*B.thetaiotaomicron* and *B.vulgatus*) that were detected in both STS and LTS. Unlike *Megasphaera* sp.*XA511*, all of them could only produce formic acid and acetic acid (**Figure 4A**), suggesting that *Megasphaera* sp. *XA511* might play a role in the stimulation of the immune system in the host through producing specific metabolites. We further co-cultured *Megasphaera* sp. *XA511* with human macrophage cell line (THP-1) to assess its immune-modulatory effect. The THP-1 cells were then separately treated with the live bacteria (*Megasphaera* sp.*XA511 LV*), heat-killed bacteria (*Megasphaera* sp. *XA511 HK*),and the secreted supernatant (*Megasphaera* sp. *XA511 SN*) to identify the actual effector. Different cytokines including IL-2, IL-6, IL-10, IFN-γ, TNF-α, IL-17A, IL-17F were quantified. We found that all treatments could enhance the production of TNF-α, while the live bacteria induced the most significant effect (**Figure 4B**), indicating that both the bacteria themselves and the supernatant composition can stimulate immune responses. Besides, both the supernatant of the bacteria and the heat-killed bacteria can stimulate the expression level of IL-6. For IL-10, the heat-killed bacteria but not the supernatant can induce its production (**Figure 4B**). These suggested that *Megasphaera* sp. *XA511 LV* could induce the cytokines production of the immune cells through the structural components of the bacteria or secretions. Other cytokines cannot be induced by the bacteria and showed undetectable abundance. To further validate its role in other immune cells, the live or heat-killed *Megasphaera* sp. *XA511* were co-cultured with the human PBMCs. Only the live bacteria can stimulate the secretion of TNF-α and IL-6 while the heat-killed cannot. And for IL-10, both the heat-killed and live bacteria can greatly promote the secretion of IL-10. These indicated that both the live and dead *Megasphaera* sp. *XA511* bacteria could stimulate the production of several kinds of pro-inflammatory cytokines in PBMCs, similar to the observations in THP1.

### *Megasphaera* sp.*XA511* Enhances the Efficacy of PD-1 Blockade *In Vivo*

**Figure 5:**
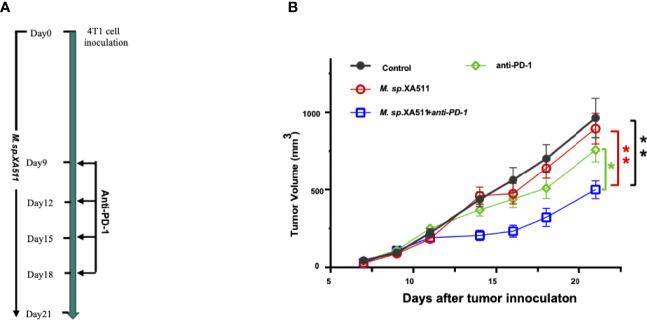
*Megasphaera* sp.*XA511 (M.sp.XA511)* enhances the efficacy of PD-1 blockade *in vivo*. **(A)** Experiment design of the mouse study. 6-week-old SPF Balb/c mice bearing 4T1 tumors were administrated with *Megasphaera* sp. *XA511* daily, Isotype or anti-PD-1 antibodies were administered to the animals on days 9, 12, 15 and 18 by intraperitoneal injection. **(B)** Tumour volumes were measured on day7, day9, day11, day14, day16, day18 and day21 after inoculation of tumour cells. Tumor size data were analysed and expressed as the mean ± standard deviation. The one-way ANOVA was used to determine whether there are any statistically significant differences between the means of PD1 combined with *Megasphaera* sp. *XA511* and other groups. The black, red, green, and blue colour indicate Control, *Megasphaera* sp. *XA511*, antiPD1 and antiPD1 combined with *Megasphaera* sp. *XA511*group respectively. The asterisk indicates significant difference (*P < 0.05, **P < 0.01).

To examine the anti-tumor effect of *Megasphaera* sp.*XA511 in vivo*, we treated the mice bearing 4T1 tumors with the *Megasphaera* sp.*XA511* strain. Although *Megasphaera* sp. *XA511* alone could not inhibit tumor growth, the combination treatment with anti-PD1 antibody, which targets programmed cell death-1 protein, significantly reduced tumor growth (**Figures 5A, B**). These results illustrate that the metabolic or other soluble factors secreted by the bacteria may be immune-stimulatory and enhance the anti-tumor effect *via* the PD1 pathway. To further explore the possible mechanisms underlying how *Megasphaera* sp. *XA511* inhibits tumor growth, multiplex panel immunofluorescence (mIHC) staining of tumor-infiltrating lymphocytes in tumor tissues from mice was performed. The density of different immune cells (CD4+ helper T cell, CD8+ cytotoxic T cell, dendric cells), as well as IFN-γ secretion cells (CD4+ IFN-γ+ positive cell and CD8+ IFN-γ+ positive cell), was quantified. Only an increase of CD4+ helper T-cells and dendric cells in some samples of PD1 blockade combined with *Megasphaera* sp.*XA511* group was observed when compared with PD1 alone or *Megasphaera* sp.*XA511* alone group (**Supplementary Figure 5**), despite the overall statistical result is not significant, due to the big variation within the same group. These suggested that the *Megasphaera* sp.*XA511* might inhibit tumor growth by influencing the tumor microenvironment, but the final inhibitory effect is a balanced result of multiple factors.

## Discussion

Previous studies have recognized intratumor microbiome as one of the risk factors to promote PDAC development, and delayed PDAC progression has been achieved in patients receiving cancer therapy combined with antibiotic treatment (39). Recently, Riquelme et al. found high tumor microbial diversity and immuno-activities in PDAC long-term survivors (LTS, median survival of 10.1 years), compared with those in PDAC short-term survivors (STS, median survival of 1.6 years) (7). However, studies have also shown that only 15% of PDAC patients can undergo surgical treatment at the time of diagnosis, with a median survival of 15 months and a 5-year survival rate of 5-7% (40). Therefore, in this study, we chose 15 months (450 days) survival time as the midpoint and defined the patients with the survival of shorter than 300 days but longer than 120 days (to exclude postoperative complications-related death) as STS, while those with survival of longer than 600 days as LTS, to investigate their microbiome composition. Interestingly, we identified higher intratumor microbiome diversity in LTS compared with STS and observed *Proteobacteria*, *Firmicutes*, and *Bacteroidetes* are the major bacteria in all samples, which is consistent with the previous report at the phylum level (7). We also found that *Sphingomonas* and *Megasphaera* were highly enriched in LTS tumor tissues, while they were not the dominant genera in the US cohort studied in Riquelme’s report (7). We wonder whether the human genetic factor from different regions influences the results, thus the microbiota profile of our Chinese cohort with that of the US was compared. The results showed that the dominant intratumor microbiota between them was significantly different although both cohorts have higher similarities in their LTS samples. For long-term and short-term survivors, the trend of difference between the US and Chinese population was the same. The microbiome of LTS samples from the same cohort was clustered together, while the two LTS groups from different cohorts were significantly different (**Supplementary Figure 6**), suggesting that such a microbial difference could be attributed to genetic differences. Different diets may also be one of the important factors as dietary can influence the microbiota in the tumor microenvironment (41, 42).

The inadequate data about the mechanism is the main limitation of this study. Instead, we focused on bacteria related to PDAC LTS samples and analyzed the microbiome differences between STS and LTS survivors. Through multiple microbial analyses, we deciphered the differential predominant phyla and genera between STS and LTS PDAC tissues and the *Megasphaera* was specifically enriched in the LTS sample. Furthermore, the *Megasphaera* sp.*XA511* isolated from a healthy donor has been shown to be advantageous in producing butyrate and other types of SCFAs over other bacteria (**Figure 4A**). SCFAs have a positive effect on cancer suppression (43–45). For example, butyrate, the most widely studied SCFA, has been considered as an inhibitor of tumor development through enhanced production of effector cytokines (46), and butyrate-mediated histone deacetylases inhibition can downregulate inflammation and dampen colorectal cancer development (47). Indeed, our *in vitro* data showed that the *Megasphaera* sp.*XA511* promotes the secretion of pro-inflammatory cytokines of THP1 as well as PBMCs. These suggested that the *Megasphaera* might enhance the immune response through the production of SCFAs. In addition, the *in vivo* experiments confirmed that the *Megasphaera* sp.*XA511* plays a significant effect on tumor suppression in the 4T1 tumor-bearing mouse model when combined with anti-PD-1 treatment. However, our current results cannot illustrate whether the oral administrated *Megasphaera* sp.*XA511* has been translocated into the mouse tumor tissues or not. More data is needed to clarify how the bacteria promote better outcomes. There are many conjectures about the mechanism. If the *Megasphaera* sp.*XA511* translocated to the tumor microenvironment, the intra-tumor bacteria could play a role in either the differentiation or proliferation of multiple tumor-infiltrating immune cells thus affecting the anti-cancer immune responses (48–50). Besides, the microbiota-derived STING agonists can also induce IFN-I production through monocytes in tumors, thereby making the tumor microenvironment more conducive for immune response (42). What’s more, the previous report has revealed that unique neoantigen quality in intratumor can be a biomarker of survival in PDAC, and microbial homology in the context of neoantigen may be a good surrogate for immunogenic neoantigens (51). Molecular mimicry of tumor neoantigens by intra-tumor microbial species might be another way in which the bacteria influence the activation of the immune response, as bacterial peptides derived from intracellular bacteria can be presented by human leukocyte antigen of tumor cells in melanoma thus eliciting immune reactivity (52, 53). Moreover, when bacteria cannot be colonized in the tumor tissue, they might affect the dendritic cells and macrophages in the intestinal lymphatic tissue (54), thus promoting the secretion of pro-inflammatory cytokines in the circulatory system.

In conclusion, our results illustrated the intratumor microbial characteristics of PDAC patients in the Chinese cohort and found that the *Megasphaera* specifically enriched in the LTS samples had a better inhibitory effect on tumor growth. The findings may offer a potential biomarker for the prediction of prognosis of PDAC patients and provide hints for further mechanistic investigation and target development of PDAC.

## Animal and Human Studies Statement

The animal study was reviewed and approved by the Institutional Animal Care and Use Committee of Shenzhen TOP Biotechnology Co., Ltd. The studies involving human participants were reviewed and approved by the China Ethics Committee of Registering Clinical Trials. The patients/participants provided their written informed consent to participate in this study.

## Data Availability Statement

The datasets presented in this study can be found in online repositories. The names of the repository/repositories and accession number(s) can be found below: NCBI BioProject Accession Number: PRJNA764032.

## Ethics Statement

The studies involving human participants were reviewed and approved by China Ethics Committee of Registering Clinical Trails. The patients/participants provided their written informed consent to participate in this study. The animal study was reviewed and approved by Institutional Animal Care and Use Committee of Shenzhen TOP Biotechnology Co., Ltd.

## Author Contributions

NZ and YY: study design. YH and XL: sample and clinical data collection. XZ, HL, and HuH: research experiments. NZ, YaL, YoL, YX and HaH: data analysis and interpretation. NZ, YaL, DW: manuscript preparation. YY, YT, and ZH: supervision, financial support, and editing. All authors contributed to the article and approved the submitted version.

## Conflict of Interest

Authors NZ, XZ, YHL, HL, XY, HJH, YT, HH, YL, and YY are employed by Xbiome Biotech Co. Ltd.

The remaining authors declare that the research was conducted in the absence of any commercial or financial relationships that could be construed as a potential conflict of interest.

## Publisher’s Note

All claims expressed in this article are solely those of the authors and do not necessarily represent those of their affiliated organizations, or those of the publisher, the editors and the reviewers. Any product that may be evaluated in this article, or claim that may be made by its manufacturer, is not guaranteed or endorsed by the publisher.

## Supplementary Material

Supplementary Table 1 Analysis of differential KEGG pathways.
