# Supplementary material for: Intratumor Microbiome Analysis Identifies Positive Association Between Megasphaera and Survival of Chinese Patients With Pancreatic Ductal Adenocarcinomas
Source: Front Immunol. 2022 Jan 25;13:785422. doi: 10.3389/fimmu.2022.785422 (PMC8821101; doi:10.3389/fimmu.2022.785422)
Supplement: Supplementary file 1 [file DataSheet_1.pdf]

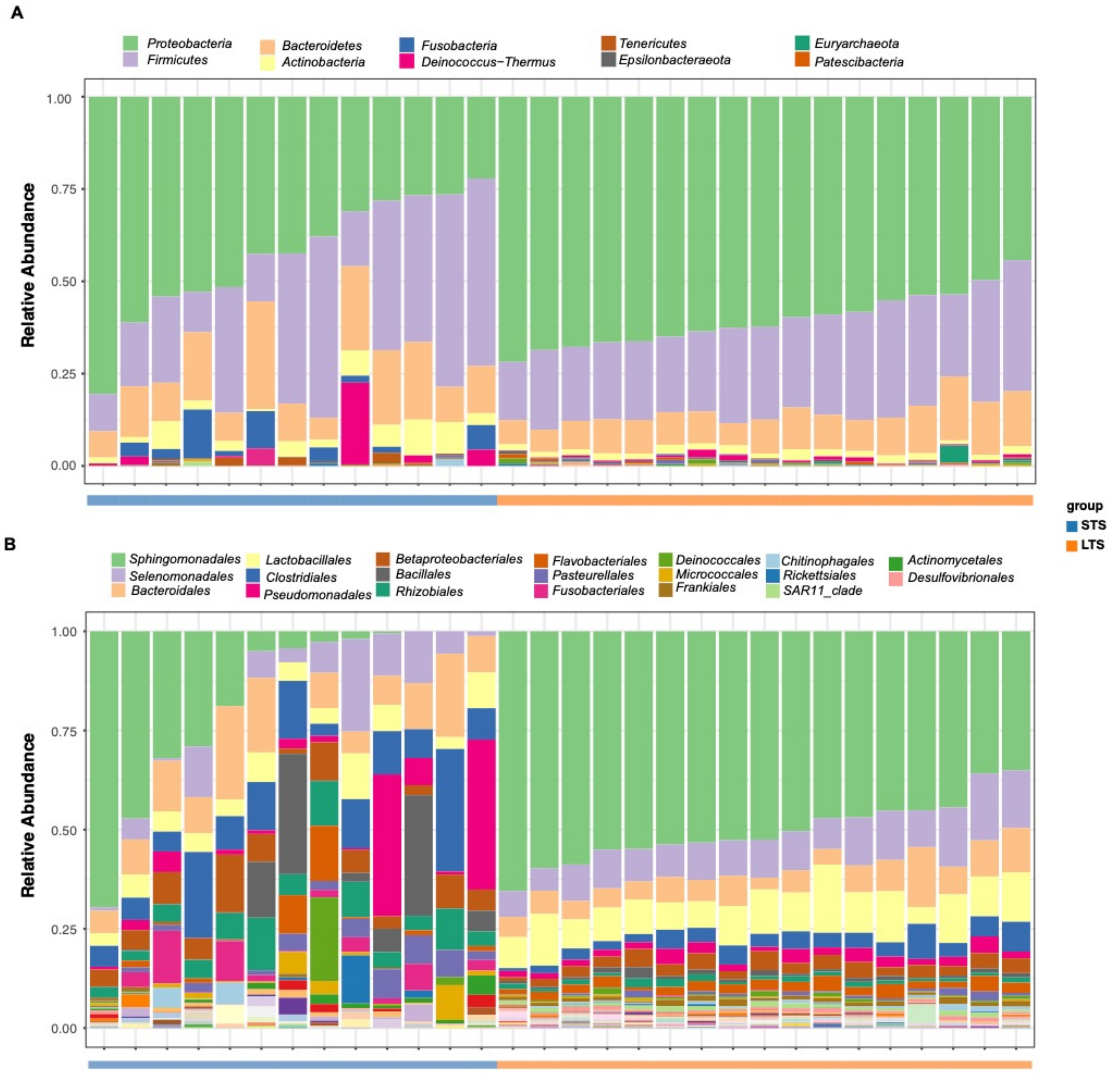

Supplementary Figure 1. Composition of LTS and STS at different levels

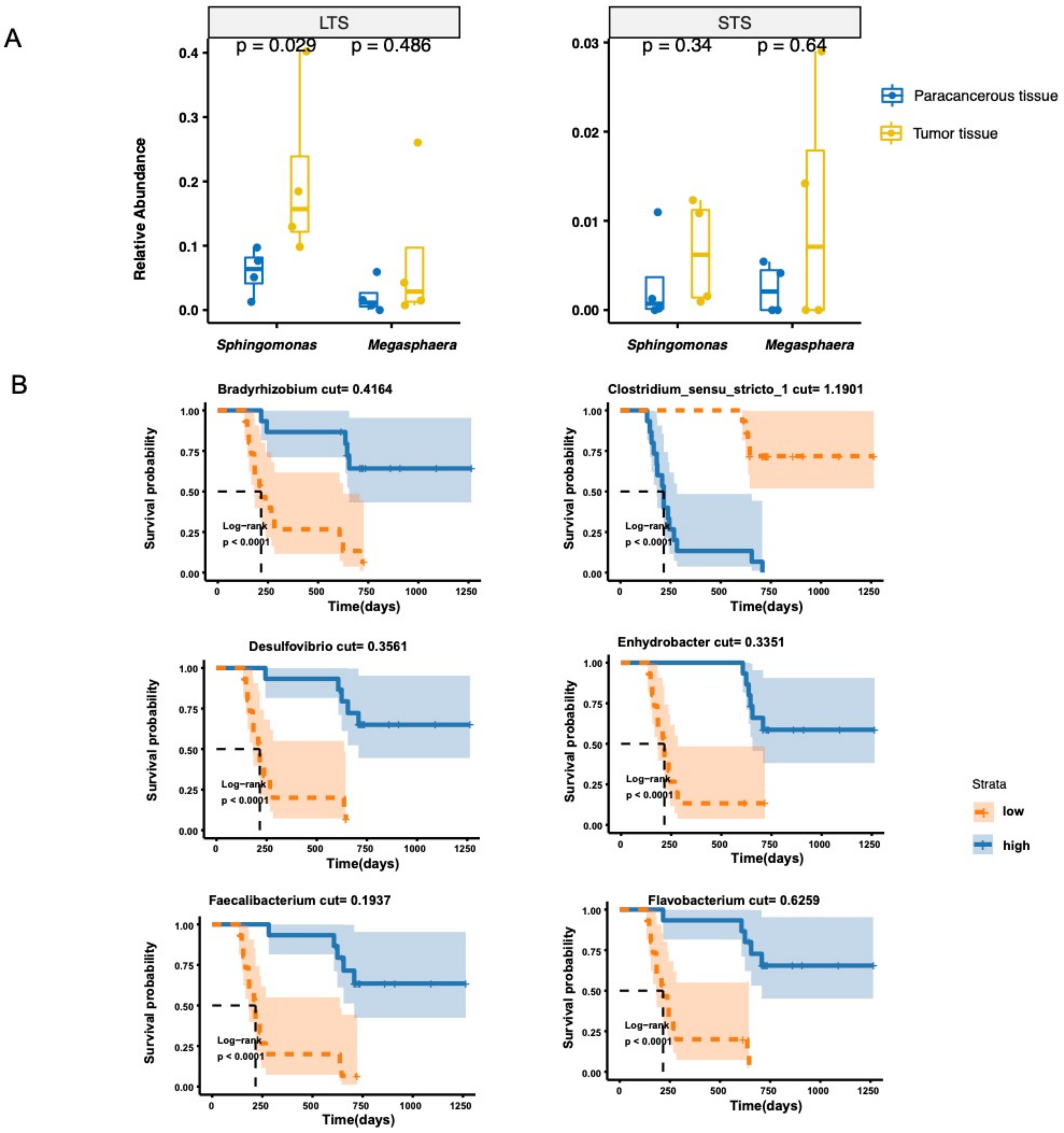

Supplementary Figure 2. (A) Relative abundance of *Sphingomonas* and *Megasphaera* in tumor and paracancerous tissue from LTS and STS (B) Kaplan-Meier estimates for survival probability with high abundance versus low abundance of different microbiome in STS and LTS patients.

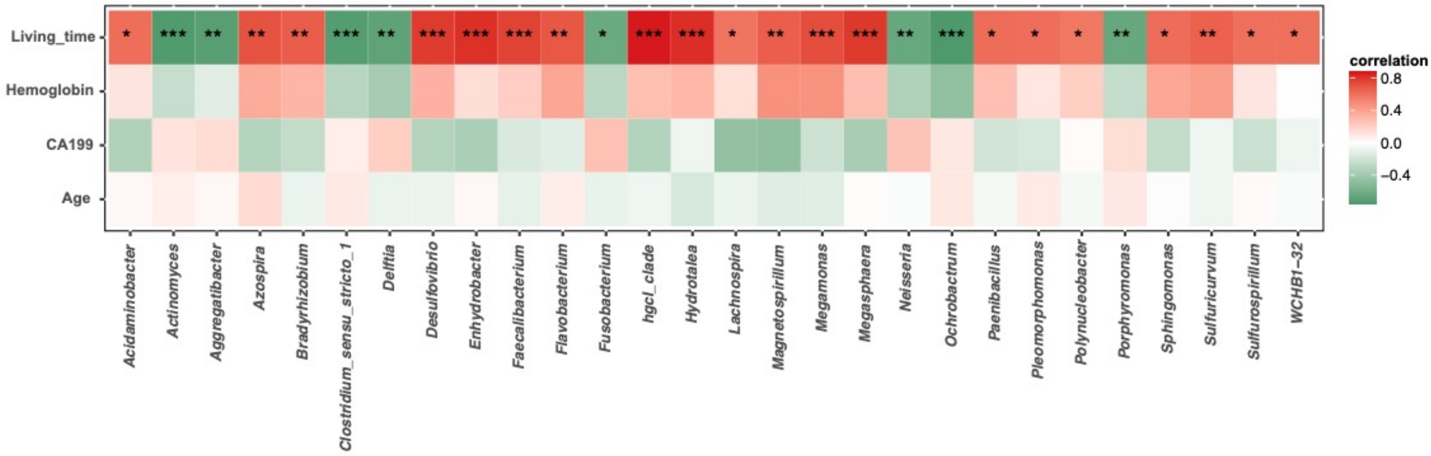

Supplementary Figure 3. Correlation of some clinical indices with microbiome genus

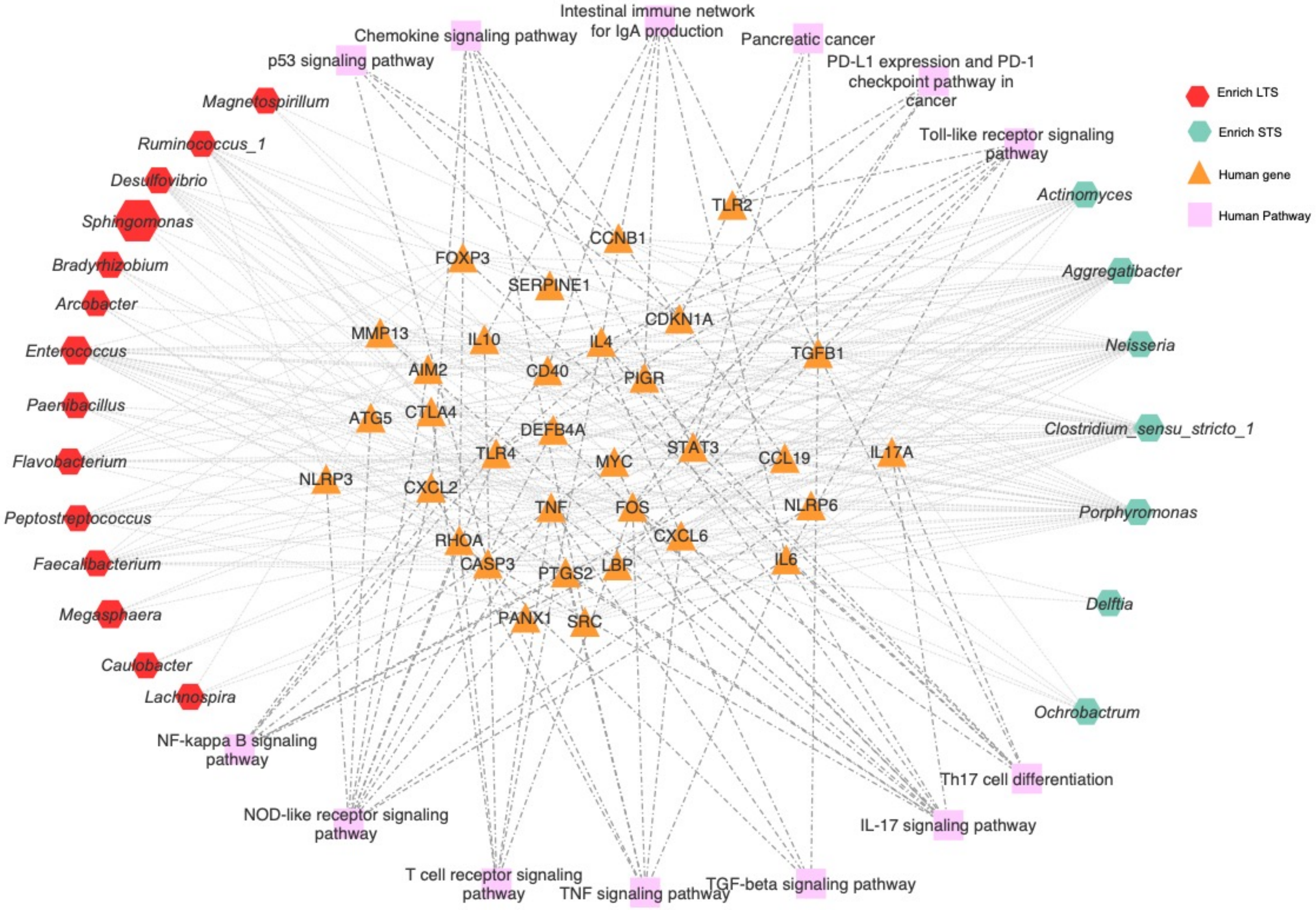

Supplementary Figure 4. Network analysis about the microbiome genus and human gene pathway by MSEA and GSEA. The red and green hexagons represent microbiome genera enriched in LTS and STS patients respectively, while the orange and purple boxes represent human genes associated with microbiomes in human enriched by MSEA. Purple boxes represent the KEGG pathway that related to those human genes by GSEA.

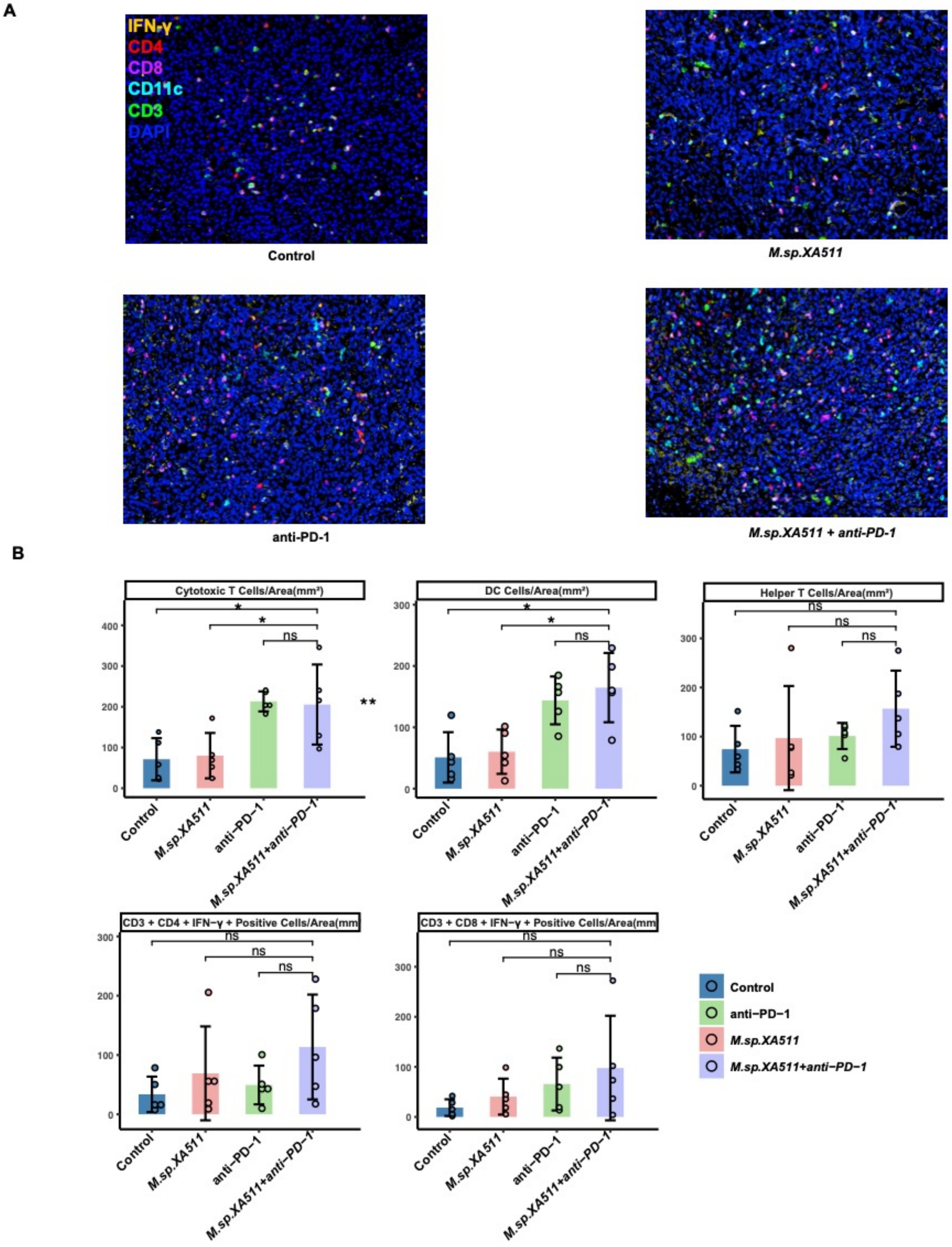

Supplementary Figure 5. The density of different immune cells and the production of IFN-γ in the tumor microenvironment of different groups. (A) Representative images of different groups Yellow= IFN-γ, green= CD3, magenta= CD8, red= CD4 and cyan= CD11c.
d and quantified by mIHC. (B) Quantification result of the whole sections with HALOTM software. The intensity of nuclear or cytoplasmic positive staining was counted. One way anova was used for statistical analysis.

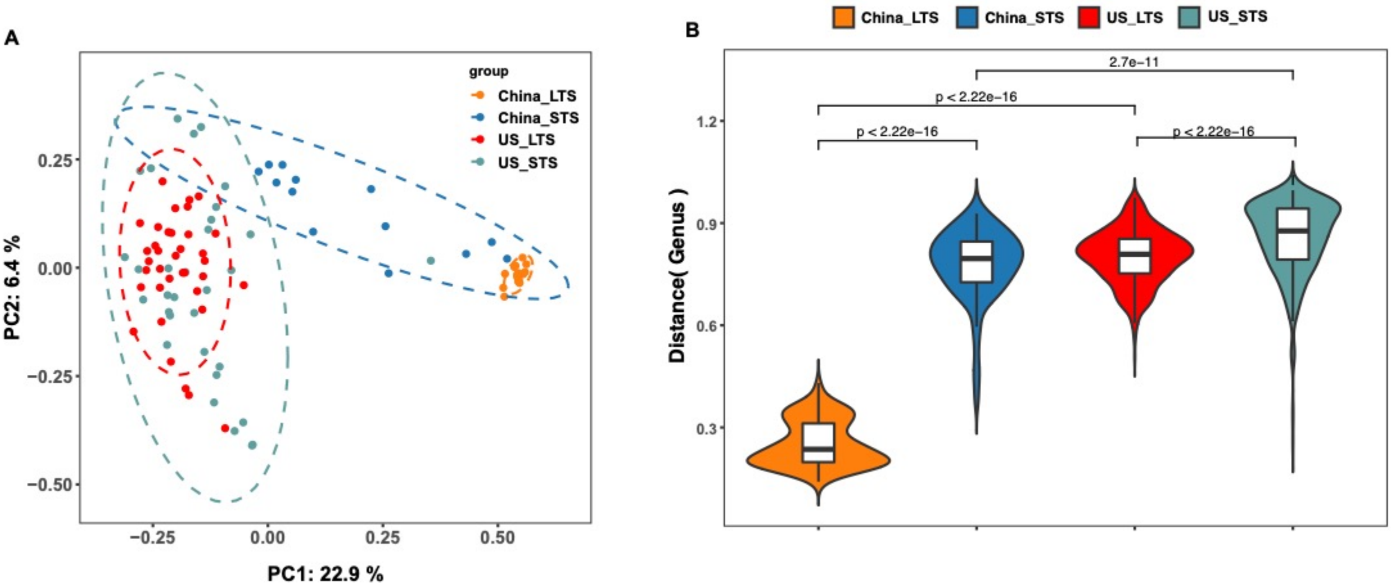

Supplementary Figure 6. Comparison of cohort between Chinese and American PDAC patients
